# Boosting 3D Object Detection with Adversarial Adaptive Data Augmentation Strategy

**DOI:** 10.3390/s25113493

**Published:** 2025-05-31

**Authors:** Shihao Li, Jingsong Li, Jianghua Fu, Qiuyue Chen

**Affiliations:** Key Laboratory of Advanced Manufacturing Technology for Automotive Parts of Ministry of Education, School of Automotive Engineering, Chongqing University of Technology, Chongqing 401320, China; lsh2023wyy@163.com (S.L.);

**Keywords:** robustness, 3D object detection, virtual adversarial training, adaptive augmentation

## Abstract

In real-world applications, autonomous driving systems need to handle a variety of complex scenarios, such as object occlusion and lighting changes. In these scenarios, accurately identifying various objects is crucial for perceiving the surrounding environment and making reliable decisions. In this context, the fusion of Lidar and cameras is vital for the accuracy of object detection. To this end, we propose an adversarial adaptive data augmentation strategy that introduces virtual adversarial perturbations during the image feature extraction process, effectively enhancing the robustness of 3D object detection methods and enabling them to maintain stable performance when facing environmental changes and data perturbations. Experimental results on the nuScenes-mini and KITTI datasets show that, compared with previous 3D object detection methods, our method not only improves detection accuracy but also demonstrates stronger stability.

## 1. Introduction

Reliable 3D object detection plays a crucial role in autonomous driving, promoting the safety and dependability of the self-driving system. Existing 3D object detection methods can be categorized into two groups: unimodal approaches and multimodal approaches. Unimodal approaches rely solely on single type of sensor for object detection, which is easy to implement but suffers inherent limitations. For example, while cameras excel in capturing rich visual details, they are susceptible to variations in ambient light and weather conditions. In contrast, Lidar provides precise three-dimensional information about objects with higher robustness to illumination variations. However, it lacks the ability to discern detailed information. Different from unimodal approaches, multimodal approaches can fully integrate both sensors to exploit their complementary information for higher accuracy and robustness. Consequently, these methods outperform unimodal ones and are widely studied in the field of 3D object detection.

In the field of multimodal 3D object detection, the key to success lies in the fusion of multimodal data. In recent years, the increasing diversity of sensor data has made the integration of complementary information from different modalities more important. Methods based on the Bird’s Eye View (BEV) [1] perspective, such as BEVDet [2] and BEVFusion [3], can effectively address issues of scale and occlusion caused by different viewpoints, enabling more efficient information aggregation and thereby enhancing the accuracy and reliability of 3D object detection. Therefore, we adopt the BEV method to transform data from different sensors into a unified top–down perspective. Despite the significant progress made by BEV methods, there are still performance bottlenecks when dealing with issues such as object occlusion.

Motivated by the great success of adversarial training, in this paper, we proposed an adaptive data augmentation method to improve the robustness of BEV-based methods. Traditional static perturbations (such as CutMix [4] and Mixup [5]) usually increase sample diversity by mixing image regions and labels, but they rely on simple linear interpolation or region mixing, which limits their improvement concerning model robustness. In contrast, our method applies small perturbations to the input images in the convergent direction. These perturbations dynamically adjust with the training process, changing their intensity and direction in real time to adapt to the model’s changes. This adaptive perturbation can continuously identify the model’s vulnerabilities and conduct targeted training, effectively preventing the model from converging prematurely. In this way, the model can learn more robust feature representations when facing different environments and data distributions. This dynamic perturbation enhances the model’s consistency in feature learning, enabling it to maintain stable performance when confronted with complex changes in real-world application scenarios. Experimental results show that our method achieves 1.5% and 0.6% improvement in terms of mean Average Precision (mAP) and nuScenes Detection Score (NDS) against the baseline on the nuScenes-mini dataset, with a 0.8% mAP improvement on the Kitti dataset, which validates the effectiveness of our method.

Our main contributions are as follows:

- We propose an adversarial adaptive data augmentation strategy (AADA) to improve the robustness of 3D object detection. To the best of our knowledge, we present the first attempt to employ virtual adversarial training for adaptive augmentation.
- Extensive experiments show that our method significantly improves the performance of 3D object detection on the nuScenes-mini and Kitti datasets, demonstrating the effectiveness of our method.

## 2. Related Work

In this section, we first briefly review Lidar-based and camera-based 3D perception methods. Then, we present recent advances in multi-sensor fusion approaches.

### 2.1. Lidar-Based 3D Perception

Due to the disordered and uneven characteristics of point clouds, existing Lidar-based 3D object detection methods commonly project point clouds into Bird’s Eye View (BEV) or Range View (RV) to utilize well-studied 2D convolutional neural networks (CNNs). In early studies, Yang et al. [6] introduced an efficient single-stage detector without anchor boxes. Graham et al. [7] proposed flat point cloud features and detected them in BEV space. Liang et al. [8] proposed employing a 2D CNN to learn spatial features from the RV, and then obtained a 3D bounding box through a Region-CNN (R-CNN). VoxelNext [9] leveraged the sparsity of point clouds to directly extract and predict 3D detection boxes based on sparse features, without converting sparse features into dense feature maps. It relies entirely on 3D CNNs, eliminating the sparse-to-dense conversion step and the NMS post-processing step. Ref. [10] proposed an innovative method that enhances the accuracy and efficiency of 3D object detection by moving point cloud features between clusters. This method performed well in complex scenarios such as occlusion and sparse point clouds, significantly enhancing the model’s discriminative ability while reducing computational costs, demonstrating its potential for application in autonomous driving. SAFDNet [11] designed an adaptive feature diffusion strategy that effectively addresses the common issue of center feature loss in sparse feature detectors and reduces the computational cost for long-range detection, improving the computational efficiency of high-performance 3D object detectors in long-range detection. Despite great progress in Lidar-based 3D object detection, existing methods still suffer from limited accuracy for distant objects due to the loss of details.

### 2.2. Camera-Based 3D Perception

As 2D object detection methods are being widely investigated and continue to improve, image-based 3D object detection methods have been developed to achieve satisfactory performance at a relatively low cost. Chu et al. [12] first performed monocular depth estimation and lifted 2D pixels to pseudo-3D points. Then, they designed a novel neighbor-voting method that incorporates neighbor predictions to improve object detection from severely deformed pseudo-Lidar point clouds. Chen et al. [13] focused on generating 3D proposals by encoding object size prior, ground-plane prior, and depth information into an energy function. Li et al. [14] introduced additional branches after the stereo Region Proposal Network (RPN) to predict sparse keypoints, viewpoints, and object dimensions, which are then used to obtain coarse 3D object bounding boxes. Guo et al. [15] leverage high-level geometric representations from LiDAR point clouds to guide the stereo image detection and introduce an auxiliary 2D detection head to provide direct 2D semantic supervision. MonoCD [16] boosted detection accuracy and robustness by leveraging the complementarity of global depth cues and geometric relationships, without enhancing the precision of individual detection branches. OPEN [17], via Object-wise Position Embedding, effectively incorporated object depth information, thereby improving the accuracy of 3D detection. Although camera-based 3D object detection methods exhibit superior results under good weather conditions, they suffer from severe accuracy drops under significant weather condition variations.

### 2.3. Multi-Sensor Fusion

To address the limitations of the aforementioned methods using only a single type of data, multi-sensor fusion has been studied to combine the best of both worlds. Early research [18] focused on sensor feature fusion. For example, a multi-camera-based joint 3D detection and segmentation method was developed using a unified BEV representation to fuse multi-camera data [19,20]. Then, subsequent research continued to improve multi-sensor fusion detection from different perspectives [21,22]. Specifically, a BEV-based multimodal fusion 3D detection method [23] was proposed to aggregate Lidar, camera, and millimeter-wave radar data with BEV for 3D object detection. Overall, it has been widely demonstrated that combining BEV with multi-sensor fusion 3D detection can improve detection accuracy and robustness.

## 3. Methodology

**Figure 1:**
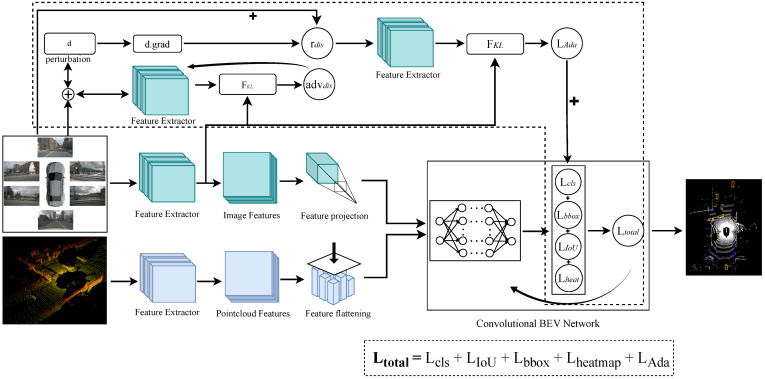
An overview of our method. Our method introduces an adversarial adaptive data augmentation strategy in the image feature extraction branch, which is denoted by the dotted box.

In this section, we propose an adversarial adaptive data augmentation strategy to enhance the robustness of multimodal 3D object detection methods. As shown in Figure 1, our network takes multiview images and Lidar point clouds as inputs. In the image feature extraction branch, the generalization ability of the model is enhanced by applying adaptive pixel-level perturbations to the images. Following this, specific encoders are employed to extract features from both images and point clouds. Then, these multimodal features are merged into a unified Bird’s Eye View (BEV) representation to reduce occlusion and eliminate viewpoint differences, facilitating the fusion process. Finally, a customized task head executes the object detection task to produce the final results.

### 3.1. Feature Extraction

#### 3.1.1. Image Feature Encoding

The input image $I_{\mathrm{img}}$ is first cropped into patches and fed to the patch embedding layer to obtain feature vectors. Then, the resultant embeddings are passed to the subsequent Transformer layers in order to integrate a self-attention mechanism to capture long-range dependencies within the image. By integrating the hierarchical features produced by each Transformer layer, multi-scale image information can be aggregated such that the network’s ability can be enhanced. In addition, a hierarchical perceptual window mechanism is also employed such that features at different levels can be concentrated in perceptual windows of different sizes, which significantly improves the efficiency of the network in processing multi-scale information.

#### 3.1.2. Point Cloud Feature Encoding

The input point cloud is first voxelized and then fed into the sparse point cloud encoder to obtain point cloud embeddings. Within the point cloud encoder, sparse 3D convolutional networks are employed for efficient point cloud feature extraction. Afterwards, the resultant sparse voxelized features are passed through the convolutional and encoder layers to generate the final features.

### 3.2. BEV Feature Transformation

To project the features of camera images and Lidar point clouds into a unified coordinate system, we construct a shared BEV plane, which effectively fuses multimodal features. This method helps to preserve geometric and semantic information while reducing efficiency bottlenecks in view transformation.

#### 3.2.1. Image-to-BEV Transformation

**Figure 2:**
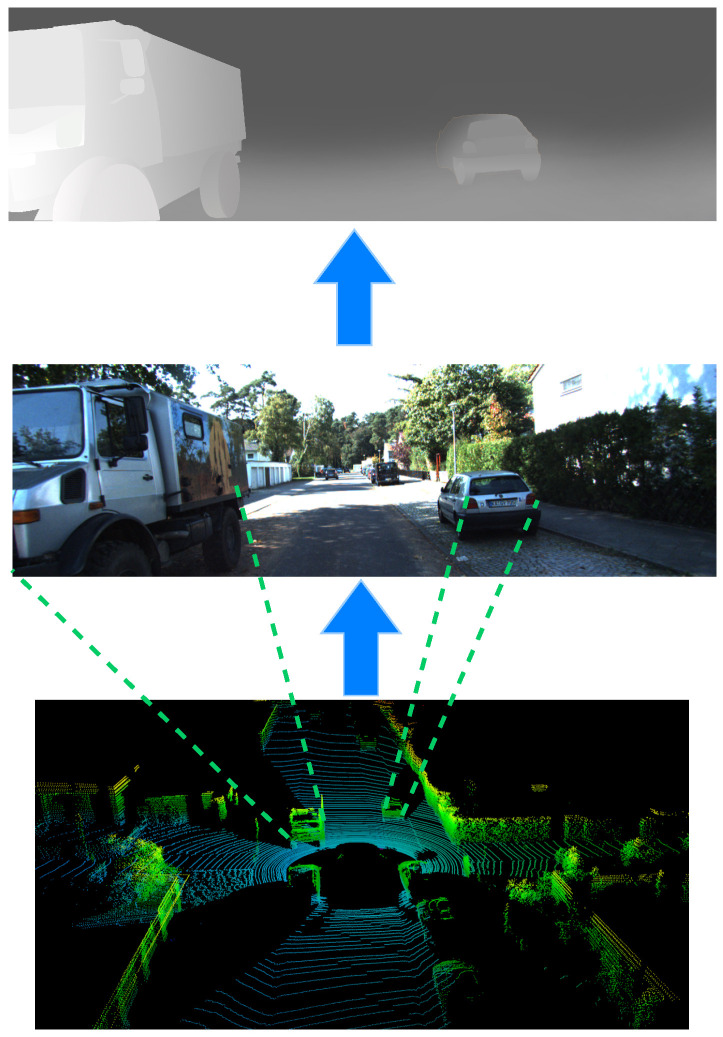
Illustration of image depth feature transformation. Point cloud is projected onto image to obtain depth information of corresponding object.

As shown in Figure 2, after projecting the Lidar point cloud onto the image, we extract the depth information of each pixel to create a depth map. Subsequently, we employ a three-layer network to extract depth features from the depth map. Then, the resultant depth features are combined with the original image features and fed to another three-layer network for feature refinement. Afterwards, a softmax layer is adopted to generate the depth distribution. Next, we multiply the depth information with the features to aggregate image and depth information. After this series of processing steps, we map the pixel coordinates (u,v) of the image depth features to the Bird’s Eye View (BEV) plane based on the extrinsic camera parameters, producing the coordinates $(x_{\mathrm{BEV}}$ and $y_{\mathrm{BEV}})$ corresponding to each pixel, as shown in Equation (1).

(1) $$\begin{array}{c} \left[\begin{array}{c} x_{\mathrm{BEV}}\\ y_{\mathrm{BEV}} \end{array}\right]=T\cdot K^{-1}\cdot \left[\begin{array}{c} u\\ v\\ 1 \end{array}\right] \end{array}$$

where *K* is the internal camera parameter matrix and *T* is the transformation matrix.

#### 3.2.2. Point Cloud-to-BEV Transformation

After point cloud feature encoding, each feature is associated with specific coordinates (x,y,z). To align these point cloud features with the BEV plane, a flattening transformation is conducted on the *z*-axis. This ensures accurate mapping of the point cloud features to their corresponding locations on the BEV plane.

### 3.3. Convolutional BEV Fusion

After converting the point cloud and image features extracted from the backbone into a unified BEV space, feature misalignment between the two modalities still hinders their fusion. To address this issue, we employ a Convolutional BEV Encoder. This encoder consists of a single-layer convolutional fusion module and the SECOND [24] network. The single-layer 3 × 3 convolutional fusion module is capable of extracting classification targets from bird’s-eye view images, effectively alleviating the local misalignment problem of multimodal features. Meanwhile, the SECOND [24] network can further extract deeper and more semantic features, enhancing the representation ability and highlighting key target information.

### 3.4. Adversarial Adaptive Data Augmentation

To enhance the generalization capability of 3D object detection methods, we propose an adversarial adaptive data augmentation strategy. By introducing minor perturbations to input images to hinder model convergence—perturbations that dynamically adapt during training—the model is trained to maintain stable predictions under these “adversarial” interferences, thereby achieving superior generalization performance.

The adversarial adaptive augmentation strategy is detailed in Algorithm 1. First, a softmax operation is conducted on $F_{\mathrm{img}}$ to obtain $F_{\mathrm{imgs}}$:

(2) $$\begin{array}{ccc} F_{\mathrm{img}} & = & f(I_{\mathrm{img}};\theta ) \end{array}$$

(3) $$\begin{array}{ccc} F_{\mathrm{imgs}} & = & softmax(F_{\mathrm{img}}) \end{array}$$

Next, we randomly generate a perturbation $d_{\mathrm{torch}}$ with the same shape of $I_{\mathrm{img}}$ and normalize $d_{\mathrm{torch}}$ to obtain $d_{\mathrm{norm}}$. $d_{\mathrm{norm}}$ is then fed into the image feature extraction module along with $I_{\mathrm{img}}$ to obtain $F_{\mathrm{imgd}}$, where the hyperparameter ξ controls the intensity of the perturbation.

(4) $$F_{\mathrm{imgd}}=\text{log\_softmax}\left(f\left(I_{\mathrm{img}}+\xi *d_{norm};\theta \right)\right)$$

  **Algorithm 1:** Adversarial Adaptive Augmentation Strategy  **Input**: $I_{img}$: Orignal image      model: Image feature extraction network      ξ: Against the size of the sample perturbation      ϵ: The size of the antagonistic sample  **Output**: $L_{Ada}$: Local adversarial loss  1  $F_{imgs}\leftarrow soft\left(model\left(I_{img}\right)\right);$  2  $d_{torch}\leftarrow create\left(I_{img}.shape\right);$ Create a tensor  3  $d_{norm}\leftarrow norm\left(d_{torch}\right);$  4  $F_{imgd}\leftarrow log_{soft}\left(model\left(I_{img}+\xi *d_{norm}\right)\right);$  5  $adv\leftarrow KL(F_{imgd},F_{imgs});$  6  adv.backward();  7  $d_{grad}\leftarrow norm\left(d_{norm}.grad\right);$  8  $r_{dis}\leftarrow d_{grad}*\epsilon ;$  9  $F_{\mathrm{dis}}\leftarrow log_{soft}\left(model\left(I_{img}+r_{dis}\right)\right);$10 $L_{Ada}\leftarrow KL\left(F_{\mathrm{dis}},F_{imgs}\right);$

Afterwards, we evaluate the difference between $F_{\mathrm{imgs}}$ and $F_{\mathrm{imgd}}$ by calculating their similarity $adv_{\mathrm{dis}}$:

(5) $$adv_{\mathrm{dis}}=\mathrm{KL}\left(F_{\mathrm{imgd}},F_{\mathrm{imgs}}\right)$$

Subsequently, we compute the gradient with respect to the perturbation $d_{\mathrm{norm}}$ and optimize $d_{\mathrm{norm}}$ to obtain $d_{\mathrm{grad}}$. $d_{\mathrm{grad}}$ is then combined with the hyperparameter ϵ to compute the adaptive perturbation $r_{\mathrm{dis}}$, where the ϵ controls the maximum perturbation value. After that, $r_{\mathrm{dis}}$ is added to the original image $I_{\mathrm{img}}$, which is then fed into the image feature extraction module to obtain the perturbed image feature $F_{\mathrm{dis}}$.

(6) $$\begin{array}{ccc} r_{\mathrm{dis}} & = & d_{\mathrm{grad}}*\epsilon \end{array}$$

(7) $$\begin{array}{ccc} F_{\mathrm{dis}} & = & \text{log\_softmax}(f\left(I_{\mathrm{img}}+r_{\mathrm{dis}};\theta \right)) \end{array}$$

Finally, we compute the KL loss between $F_{\mathrm{imgs}}$ and $F_{\mathrm{dis}}$ as the adaptive perturbation loss $L_{\mathrm{Ada}}$:

(8) $$L_{\mathrm{Ada}}=\mathrm{KL}\left(F_{\mathrm{dis}},F_{\mathrm{imgs}}\right)$$

### 3.5. Loss Function

In our model, the total loss function $L_{total}$, as shown in Equation (13), consists of the following components: The classification loss $L_{cls}$ is used to evaluate the model’s classification accuracy by reflecting the differences between the predicted and ground-truth classes. The IoU loss $L_{IoU}$ measures the overlap between the predicted and ground-truth bounding boxes, and optimizing it can make the predicted boxes more accurately cover the targets. The bounding box loss $L_{bbox}$ calculates the coordinate deviations between the predicted and ground-truth bounding boxes, helping to precisely adjust the position and size of the boxes. The keypoint detection regression loss $L_{heat}$ ensures the accurate positioning of keypoints. In addition, we introduce the adaptive augmentation loss $L_{Ada}$ to enhance the model’s robustness in different environments. The combined effect of these loss functions drives the model to achieve high accuracy and stability in the object detection task.

(9) $$\begin{array}{ccc} L_{cls} & = & -\alpha_{t}{\left(1-p_{t}\right)}^{\gamma } \end{array}$$

(10) $$\begin{array}{ccc} L_{IoU} & = & -\log\left(p_{t}\right)+\log\left(\sigma \left(p_{t}\right)\right) \end{array}$$

(11) $$\begin{array}{ccc} L_{bbox} & = & \sum_{i=1}^{4}\left|t_{pred,i}-t_{gt,i}\right| \end{array}$$

(12) $$\begin{array}{ccc} L_{heat} & = & \sum_{i}-\alpha_{t}{\left(1-p_{t}\right)}^{\gamma }e^{-\frac{{\left(d_{i}-\mu_{i}\right)}^{2}}{2\sigma^{2}}} \end{array}$$

(13) $$\begin{array}{ccc} L_{total} & = & \alpha_{1}\cdot L_{cls}+\alpha_{2}\cdot L_{IoU}+\alpha_{3}\cdot L_{bbox}+\alpha_{4}\cdot L_{heat}+\alpha_{5}\cdot L_{Ada} \end{array}$$

where $p_{t}$ is the predicted probability, $\alpha_{t}$ is a weighting factor, γ is an adjustment factor, $\sigma \left(p_{t}\right)$ is used to adjust the weights in the loss function. $t_{pred,i}$ and $t_{gt,i}$ are the parameters of the predicted bounding box and the true bounding box, and $\alpha_{*}$ denotes the weighted value of the loss.

## 4. Experiments

### 4.1. Datasets

To evaluate its effectiveness, we conducted experiments on the nuScenes-mini and KITTI [25] datasets. The nuScenes-mini dataset is a subset of the nuScenes [26] dataset, including ten scenes and 1000 samples. The mean average precision (mAP) and nuScenes detection score (NDS) are used to evaluate and compare the performance of different methods. The KITTI dataset provides 14,999 images and corresponding point clouds for the detection task, of which 7481 groups are used for training and 7518 groups are used for testing. The dataset is divided into three categories: easy, medium, and difficult according to whether the annotation box is occluded, the degree of occlusion, and the box height. The mAP is used to evaluate different methods.

### 4.2. Implementation Details

On the nuScenes-mini dataset, we set ξ to 5, ϵ to 2, epoch to 12, and $\alpha_{1}$ and $\alpha_{2}$ to 1 and 0.1, respectively. The batch size was set to 4, the minimum learning rate was set to 0.0001, and a cluster equipped with four V100 GPUs was used for training. On the KITTI dataset, we used the same training parameters as the nuScenes-mini dataset. All experiments were conducted on a PC with four NVIDIA V100 GPUs.

### 4.3. Evaluation Results

#### 4.3.1. Results on nuScenes-Mini Validation Set

A series of state-of-the-art 3D object detection methods were re-implemented for evaluation, including PointPillars [27], PGD [28], Centerpoint [29], DAL [30], UVTR [31], Sparsefusion [32], and BEVFusion [3]. As shown in Table 1, the number of training iterations was adjusted during re-implementation to ensure fair comparison. Detection results on the nuScenes-mini validation set are reported in Table 1, where our method is observed to achieve a 1.5% improvement in mAP and a 0.4% improvement in NDS compared to the baseline detector BEVFusion. Furthermore, superior performance over previous approaches is demonstrated, confirming the method’s effectiveness. Increased computational resources are limited to 14% relative to BEVFusion, with training time per epoch being extended by only 47 s, as indicated in Table 2.

#### 4.3.2. Results on KITTI Validation Set

To further verify the effectiveness of the proposed method, additional experiments were conducted on the KITTI dataset. MVXNet [33] was selected as the baseline model, with the original image feature extraction network (ResNet [34]) being replaced by Swin Transformer [35]. The proposed AADA strategy was subsequently introduced to enhance model robustness. Experimental results are presented in Table 3.

As indicated in the results, the proposed method achieves a minimum improvement of 0.8% in mean average precision (mAP). Of particular significance is the observed performance enhancement in cyclist detection tasks, where superior results are demonstrated compared to the baseline detector across varying difficulty levels, with respective improvements of 3.4%, 2.6%, and 1.7% being achieved. These findings not only validate the method’s effectiveness, but also reveal its capability to consistently improve detection performance under different challenge conditions, particularly in the more demanding scenario of cyclist detection. The demonstrated improvements are considered to provide substantial evidence for the method’s practical application potential.

### 4.4. Ablation Study

This section analyzes the specific effects of hyperparameters ξ and ϵ on method effectiveness, with particular focus on their impact on model performance. Ablation experiments are subsequently conducted through systematic comparison between the proposed adversarial perturbation and Gaussian noise.

#### 4.4.1. Impact of ϵ

The hyperparameter ϵ determines the maximum perturbation magnitude during adversarial sample generation. Smaller ϵ values result in generated samples being closer to the original inputs. However, excessively small ϵ may compromise model robustness against real-world perturbations. Experimental investigation of ϵ values (Table 4) reveals optimal performance with ϵ = 2, where the proposed method achieves peak mAP and NDS scores. This configuration is consequently adopted as the default experimental setting.

#### 4.4.2. Impact of ξ

Unlike ϵ, ξ directly controls the magnitude of the input perturbation during training, which is proportional to ξ. By appropriately increasing the input perturbation, the model is prone to be more robust to perturbations. Nevertheless, if ξ is too large, excessive perturbation may negatively affect the model performance. Consequently, we conduct experiments to investigate the effects of ξ and present the results in Table 5. As we can see, our model achieves the best performance in terms of mAP and NDS when ξ is set to 5. This suggests that by carefully tuning the ξ parameter, the model’s ability to adapt to input perturbations can be enhanced while maintaining its performance.

#### 4.4.3. Comparison with Other Data Augmentation Methods

To highlight the superiority of our method, we conducted comparative experiments with baseline models and multiple perturbation strategies under specific experimental conditions. In the experiments, we selected the following three perturbation types: Gaussian noise, viewpoint transformation, and the dynamic data augmentation strategy PGD [36]. Gaussian noise was chosen as a common perturbation form due to its broad applicability and effective perturbation performance, with its mean and standard deviation set to 0 and 25, respectively, to ensure reasonable and effective perturbations. The viewpoint transformation method simulates image variations under different perspectives by adjusting angles and dimensions, where the rotation angle range is set between −5.4 to 5.4 degrees, and the scaling ratios for height and width are within 0.38 to 0.55 times, mimicking potential viewpoint changes encountered during real-world vehicle operation. For the PGD strategy, the perturbation norm is 0.015, the perturbation step size is 0.01, and the number of iterations is set to 10, ensuring controllability of perturbations and reproducibility of experiments. The experimental results are shown in Table 6.

As shown in the table, our method demonstrates significant advantages over static data augmentation approaches—Gaussian noise and view transformation. In comparison with the dynamic data augmentation strategy PGD, although our method exhibits a slightly lower mAP metric than PGD, the training time of PGD is approximately twice as long as that of our method. This indicates that our approach achieves high performance while substantially reducing training time costs, demonstrating superior efficiency and practical applicability.

### 4.5. Visualization Results

**Figure 3:**
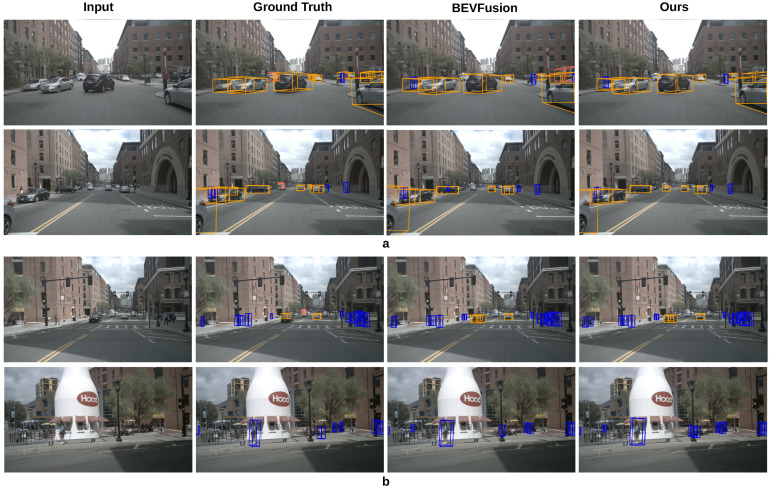
Object detection results for our method on the nuScenes-mini dataset. (**a**,**b**) demonstrate that our method produces superior results when dealing with occlusions and unlabeled objects.

**Figure 4:**
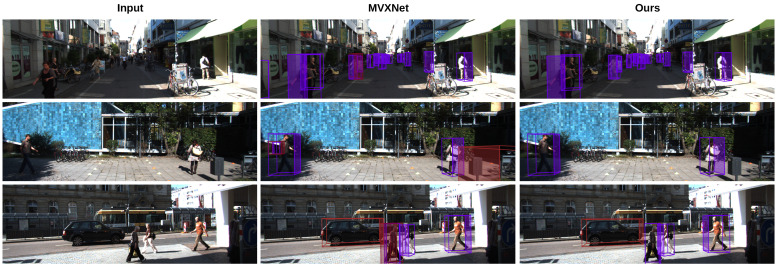
Object detection results of our method on the KITTI dataset.

In Figure 3, we present 3D object detection results achieved on the nuScenes-mini dataset. As we can see, our method can effectively identify objects even in challenging scenes, such as occluded objects and close objects. As shown in Figure 3a, our method captures details that may be overlooked in natural scenes. In addition, as shown in Figure 3b, our method is also able to recognize objects without explicit labels. In Figure 4, we can see that our method has better recognition effect than MVXNet, which further demonstrates the effectiveness of our method.

## 5. Limitations and Future Research

### 5.1. Limitations

Despite the superior performance, our approach still has some challenges.

#### 5.1.1. Poor Recognition of Small Objects

**Figure 5:**
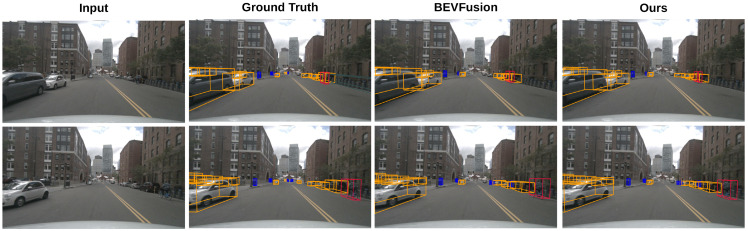
Object detection results for our method on the nuScenes-mini dataset.

As shown in Figure 5, when objects are distant and small, their recognition becomes more challenging. This is primarily because these small, far-away objects occupy only a few pixels in the image. Our feature extraction network partitions the image into several non-overlapping local windows for self-attention computation. The division of local windows makes feature extraction in each window relatively independent, with limited information interaction across windows. Small targets may be scattered across multiple windows, making it difficult for the model to capture their global features and contextual information, which affects the accurate detection of small targets.

#### 5.1.2. Limitations of Manual Hyperparameter Selection for AADA

The two key hyperparameters of AADA (perturbation size and number of perturbed samples) significantly affect the algorithm’s performance. Manually setting these parameters has limitations because they are difficult to adapt to different datasets and task requirements, necessitating extensive experiments for tuning, which is a cumbersome and time-consuming process. Moreover, the manually chosen parameters may no longer be applicable when the data distribution or environment changes, leading to unstable algorithm performance.

### 5.2. Future Research Trends

In response to the limitations of the current algorithm, our future work will focus on the following aspects:

1. Enhancing small-object extraction capability: We will consider dynamically adjusting the size and position of local windows in the image feature extraction network based on image content, focusing on feature extraction in small-object regions. Meanwhile, we will design feature extraction modules tailored for small objects to increase the model’s sensitivity and extraction efficiency for small-object features.
2. Diverse environment evaluation: We will conduct comprehensive environmental adaptability assessments of the proposed method. The performance of the method will be tested under various environmental conditions, including night driving and adverse weather, to analyze its limitations and optimize the algorithm in a targeted manner.
3. Automated hyperparameter tuning: We will develop automated hyperparameter adjustment methods to efficiently explore the hyperparameter space and identify better parameter combinations, thereby improving the overall performance and stability of the algorithm.

## 6. Conclusions

This paper proposes an adversarial adaptive data augmentation strategy to enhance the robustness of 3D object detection. Unlike traditional data augmentation methods that rely on simple linear interpolation or image region mixing, our AADA method introduces adaptive perturbations during training that can dynamically adjust their intensity and direction in real time to adapt to model changes and prevent premature convergence. These perturbations can identify model weaknesses and conduct targeted training, prompting the model to generate more consistent outputs, thereby achieving superior performance. Experimental results on the nuScenes-mini and KITTI datasets demonstrate the significant effectiveness and practicality of our method in 3D object detection. 

## Figures and Tables

**Table 1:** Performance comparison on the nuScenes-mini validation set.

Method	mAP	NDS	AP					
			Car	Truck	Bus	Ped.	Motor	Bicycle
PointPillars	29.6	40.8	83.5	31.2	75.1	77.5	7.0	21.4
PGD	30.7	32.3	52.0	44.2	52.8	45.3	35.1	19.3
CenterPoint	37.3	46.6	80.3	60.7	86.6	87.8	17.2	-
DAL	44.9	53.4	85.7	59.4	64.9	91.4	38.8	12.3
UVTR	42.9	50.0	86.7	63.5	94.7	85.4	41.1	4.0
Sparsefusion	49.3	47.0	85.4	**72.5**	72.9	91.8	54.0	**34.4**
BEVFusion	48.1	52.9	**89.2**	64.1	98.2	91.8	56.0	27.7
Ours	**49.6**	**53.3**	88.3	69.1	**99.3**	**92.1**	**58.8**	33.4

**Table 2:** Comparison of training efficiency on the nuScenes-mini dataset.

Method	Training Time per Epoch(s)	GPU Memory (MIB)
BEVFusion	216	17,847
Ours	263	20,410

**Table 3:** Performance comparison on the KITTI validation set.

Method	mAP	Car			Pedestrain			Cyclist		
	Mod.	Easy	Mod.	Hard	Easy	Mod.	Hard	Easy	Mod.	Hard
MvxNet-ResNet	63.5	88.4	**78.8**	74.7	62.9	58.4	**55.2**	70.9	53.2	49.6
MvxNet-SwinT	64.1	88.2	78.2	75.5	64.1	**58.5**	54.1	71.9	55.6	52.7
Ours	**64.9**	**89.5**	78.3	**75.7**	**64.7**	58.0	53.6	**75.3**	**58.2**	**54.4**

**Table 4:** Comparison of 3D object detection performance on the nuScenes-mini dataset with different ϵ values.

ξ	ϵ	mAP	NDS	AP					
				Car	Truck	Bus	Ped.	Motor	Bicycle
5.0	1.0	47.9	52.3	**88.6**	64.0	97.3	90.4	54.7	28.3
5.0	2.0	**49.6**	**53.5**	88.3	**69.1**	**99.3**	**92.1**	**58.8**	33.4
5.0	3.0	48.7	53.1	**88.6**	65.9	98.3	91.3	54.6	**35.1**

**Table 5:** Performance comparison of 3D object detection on the nuScenes-mini dataset with different ξ values.

ξ	ϵ	mAP	NDS	AP					
				Car	Truck	Bus	Ped.	Motor	Bicycle
3.0	2.0	48.8	52.2	88.4	63.4	99.2	91.5	47.7	**39.2**
4.0	2.0	48.8	52.6	88.1	66.6	98.3	91.4	48.6	36.2
5.0	2.0	**49.6**	53.5	88.3	**69.1**	99.3	**92.1**	**58.8**	33.4
6.0	2.0	48.1	52.9	88.3	67.0	99.3	91.0	50.4	34.8
7.0	2.0	48.8	53.0	88.4	65.0	97.9	91.2	54.7	35.4
8.0	2.0	48.6	53.2	88.3	67.0	97.9	91.2	57.9	30.2
9.0	2.0	49.1	**53.7**	**88.6**	69.0	99.3	91.0	56.5	29.1
10.0	2.0	49.3	52.9	88.2	67.8	**99.4**	91.8	51.8	36.1

**Table 6:** Comparison of 3D object detection results with different types of perturbation.

Method	mAP	NDS	AP					
			Car	Truck	Bus	Ped.	Motor	Bicycle
Based on Gaussian noise	49.0	53.2	88.8	66.9	**99.7**	91.6	57.5	36.3
Based on view transformation	48.1	52.9	**89.2**	64.1	98.2	91.8	56.0	27.7
Based on PGD	**50.2**	53.1	88.6	68.0	99.5	91.5	**58.9**	**37.8**
Ours	49.6	**53.5**	88.3	**69.1**	99.3	**92.1**	58.8	33.4

## Data Availability

Data are contained within the article.
